# Critical care rotation impact on pediatric resident mental health and burnout

**DOI:** 10.1186/s12909-017-1021-1

**Published:** 2017-10-05

**Authors:** Katie K. Wolfe, Sharon M. Unti

**Affiliations:** Division of Critical Care Medicine, Department of Pediatrics, McGaw Medical Center/Northwestern University Feinberg School of Medicine, 225 E. Chicago Ave, Chicago, IL 60611 USA

**Keywords:** Resident, Burnout, Depression, ICU

## Abstract

**Background:**

Burnout and depression are common among medical trainees and intensive care unit providers, negatively impacting both providers and patients. We hypothesized that at the end of the pediatric intensive care unit (PICU) rotation, there would be an increased prevalence of depression and burnout in pediatric residents when compared to the beginning.

**Methods:**

Pediatric residents were assessed prior to and following their PICU rotation using the Maslach Burnout Inventory, the Center for Epidemiologic Studies Depression Screen and a survey assessing positive and negative aspects of the rotation.

**Results:**

Sixty residents were eligible to participate and initial response rate was 40%. The prevalence of positive depression screen increased from 4% to 41% during the PICU rotation. Regarding burnout, the prevalence of residents meeting criteria for emotional exhaustion increased from 41% to 59% and depersonalization increased from 41% to 53%. Fewer residents had low personal accomplishment scores at the end of the rotation, 13% to 0%. Autonomy, procedural opportunities, and interactions with non-trainee PICU providers were commonly cited negative aspects of the rotation. Resident education, patient acuity, and nursing-integrated rounding were consistently rated positively.

**Conclusion:**

Compared to the beginning, at the end of the PICU rotation there is a significantly higher prevalence of depression, emotional exhaustion, and depersonalization among pediatric residents. Pediatric residents may have a more favorable PICU experience if they feel involved in procedural aspects of patient care, are allowed more autonomy in decision making, and there is a continued focus on resident education and team-based care.

## Background

Burnout is a maladaptive syndrome characterized by emotional exhaustion, depersonalization and decreased feelings of personal accomplishment, typically associated with chronic work stress [1]. Resident physicians are at high risk of burnout, with current literature demonstrating prevalence of 50–75% in this population [2–10]. Burnout and depression are often coincident; depressive symptoms are present in 50–90% of residents meeting criteria for burnout [5, 6, 9, 11, 12]. In addition to the personal impact burnout and depression have on residents, evidence suggests they can negatively impact patient care [9]. One study of pediatric residents found that depressed residents were five times more likely to make medication errors [5].

The Pediatric Intensive Care Unit (PICU) rotation is an important, yet challenging, educational experience during a pediatric resident’s training. The high patient volume and acuity, long hours, and other demands inherent to the rotation can result in a significant amount of stress placed on residents. Attending physicians and nurses in the ICU are known to experience high rates of burnout and depression which is likely to impact those residents whom they are teaching [13–17]. The resident experience in the PICU, and its impact on resident well-being, has not been extensively studied. We hypothesized that pediatric residents would experience higher rates of depression and burnout in at least one domain at the end of their critical care rotation compared to the beginning.

## Methods

### Setting and participants

We conducted a prospective study, assessing post-graduate year-2 pediatric residents at McGaw Medical Center/Northwestern University Feinberg School of Medicine rotating in the PICU at Ann & Robert H. Lurie Children’s Hospital of Chicago over a 13 month period (December 2014–December 2015). The study was reviewed by the IRB and considered exempt. Residents were surveyed on the first day of their PICU rotation and immediately following the 4-week rotation. Non-pediatric residents were excluded from participation. Gender and first vs. second PICU rotation were the only demographic data collected. At the time of the study, the 40-bed PICU was divided among two teams; one pediatric resident-staffed team with a pediatric critical care fellow and attending, and one advanced practice nurse and pediatric hospitalist-staffed team with a fellow and attending physician. On average, the resident team typically cared for 14–20 critically-ill patients (3–5 patients per resident) and residents took 24 h call every 4–5 days. The non-resident team typically cared for 10–14 patients and was staffed on a day/night shift schedule. Also during this study, nursing-integrated rounds; during which bedside nurses presented objective data including sedation drips, vasoactive medications, and ventilator settings, were started in the PICU.

### Data collection and instruments

Participation was voluntary on the part of the residents. Paper copies of the surveys were distributed to the participants and were labeled with an anonymous participant identifier. Surveys took less than 10 min to complete and were completed by residents within the first 2 days of their PICU rotation. Post-rotation surveys were only completed by residents who completed the pre-rotation surveys.

The Maslach Burnout Inventory is a 22-question tool scored in 3 domains; emotional exhaustion, depersonalization and personal accomplishment [1]. It has been validated in adults in a variety of professions but not in pediatric residents specifically. In the domain of emotional exhaustion, average scores are typically 19–26 with positive score being greater than 26. For questions related to depersonalization, a score of greater than 9 is considered high. Average scores for personal accomplishment are 34–39 with a positive result being less than 34. Residents were considered to meet criteria for burnout if they scored positively in at least one of the three domains.

The Center for Epidemiologic Studies Depression Scale (CES-D) was used to assess risk of depression [18]. This is a 20 question screening tool. Scores of less than 15 indicate no depression, 15–20 mild to moderate depression and greater than or equal to 21 are concerning for possible major depression. This tool has been validated in a general adult population.

We also administered an 8-question survey assessing the anticipated (pre-rotation) or perceived (post-rotation) positive and negative aspects of the experience. This survey was scored on a 4-point Likert scale (very positive, positive, negative or very negative) and asked questions regarding the overall experience, patient acuity, workload, education, procedural opportunities, autonomy, interactions with non-trainee PICU team and the institution of nursing-integrated rounds. A copy of the survey is available in the appendix.

### Outcomes

The primary outcomes for this study were prevalence of depression and burnout (in at least one domain) among surveyed residents at the beginning and end of their PICU rotation. Secondary outcomes included data gathered from the 8-question survey; positive and negative aspects of the PICU experience.

### Statistical analysis

Results of the 3 domains of the Maslach Burnout Inventory and CES-D were analyzed as categorical variables, using the thresholds identified as positive or negative screens for the validated tools (described in the data collection and instruments section), reported as percentages and compared using McNemar’s test, with a *p* value of <0.05 considered statistically significant. The results of the 8-question survey were also treated as binary categorical variables (positive or negative) and reported as percent positive responses. Data from prior to and following the rotation were compared using McNemar’s test*.* The total number of data points was too small to complete regression models associating positive and negative aspects of the experience with depression or burnout. Analysis was completed using IBM SPSS Statistics (2015).

## Results

Approximately 60 residents were eligible to participate in this study over the one-year study period. All participants were second-year pediatric residents. Twenty-four residents completed the pre-rotation surveys and 17 also completed the post-rotation surveys. The response rate for pre-rotation surveys was 40% and 70% of those residents also completed post-rotation surveys. Four participants were male. Nine residents were surveyed during their first PICU rotation with the remaining 15 residents completing surveys during their second PICU rotation.

Primary outcomes are summarized in Table 1. The rate of positive depression screen (CES-D score > 15) was significantly increased at the end of the PICU rotation compared with the beginning (7 of 17 (42%) vs 1 of 24 (4%) *p* < 0.001). Ten of 24 (41%) residents met criteria for burnout in at least one domain at the beginning of the rotation compared with 11 of 17 (65%) residents at the end of the rotation. In the domain of emotional exhaustion, significantly more participants met criteria at the end of the rotation; 10 of 17 (59%), compared with the beginning, 10 of 24 (41%). Similarly, in the domain of depersonalization, significantly more residents met criteria at the end of the rotation, 9 of 17 (53%), when compared to the beginning of the rotation, 10 of 24 (41%). Personal accomplishment scores remained high prior to and following the rotation. 3 of 24 residents (12.5%) had low personal accomplishment scores prior to the rotation and no residents had low scores (<34) following the rotation. This decrease in low personal accomplishment scores was statistically significant.

**Table 1:** Comparison of prevalence of depression and burnout prior to and following the PICU rotation

	Pre-rotation (*n* = 24)	Post-rotation (*n* = 17)	*p* value
Depression	1 (4)	7 (42)	< 0.001
Burnout			
Emotional exhaustion	10 (41)	10 (59)	< 0.001
Depersonalization	10 (41)	9 (53)	0.024
Personal Accomplishment	3 (13)	0 (0)	< 0.001

Results are reported as total number who screened positive with percentage of total group in parentheses; n (%)

Secondary outcomes are summarized in Table 2. We compared the proportion of residents responding “positive” or “very positive” to questions regarding the PICU rotation and potential influential aspects of the experience. Prior to the rotation 91% of residents anticipated it to be a positive experience and 82% of residents reported a positive experience at the end. This change was statistically significant. Overall workload and autonomy were more frequently rated as positive aspects of the rotation following the experience (63% to 71% for workload and 58% to 75% for autonomy). These changes were also statistically significant. Residents at the end of the rotation reported less positive perceptions regarding the opportunity to do procedures and interactions between the two PICU teams. Patient acuity, nursing integrated rounding and overall resident education were consistently rated as positive aspects of the PICU rotation, both prior to and following the experience.

**Table 2:** Comparison of participants answering “positive” or “very positive” to questions regarding anticipated (pre-rotation) or perceived (post-rotation) aspects of the PICU experience

Aspect of PICU Rotation	Pre-rotation (*n* = 24)	Post-rotation (*n* = 17)	*p* value
Overall Experience	22 (91)	14 (82)	0.020
Patient Acuity	23 (96)	16 (94)	0.028
Resident Workload	15 (63)	12 (71)	< 0.001
Resident Autonomy	14 (58)	11 (65)	< 0.001
Opportunity to do procedures	21 (88)	9 (56)	< 0.001
Interactions between PICU teams	19 (79)	11 (65)	< 0.001
Nursing Integrated Rounding	24 (100)	16 (94)	0.230
Resident Education	24 (100)	16 (94)	0.230

Results are reported a total number with percentage of total group in parentheses; n (%)

## Discussion

Pediatric residents have a significantly increased incidence of depression, depersonalization and emotional exhaustion at the end of their PICU rotation. The increased incidence of depression is most marked. While the incidence of burnout was high among residents at the beginning of the rotation, indicating that pediatric residents experience burnout throughout training, it significantly increased during the PICU experience. Interestingly, residents had high scores in the domain of personal accomplishment and this increased during the PICU rotation. This may indicate that despite the challenges inherent to the training experience in the PICU, residents do feel they are making a difference and growing as physicians.

The primary strength of this study is the description of burnout and depression among pediatric residents, specifically during the PICU experience. Perceived positive and negative aspects of the PICU rotation were assessed to inform potential changes or interventions to the PICU experience for residents. This survey was also helpful in identifying things about the PICU culture that were working well and should be encouraged. Education, nursing involvement in rounds and the exposure to high acuity patients were consistently perceived as positive components of the PICU experience and should be encouraged and developed further. While anticipated autonomy was considered a negative prior to the rotation, perceived autonomy increased at the end of the rotation. This reinforces more active engagement of residents in decision making and patient care.

Another aspect which could be improved upon is the procedural experience for residents in the PICU. Due to the presence of many fellows in the PICU, with various levels of procedural experience, residents infrequently preform procedures themselves. However, residents also feel uninvolved in the procedures occurring for their patients, which can be changed. Having residents observe and assist with procedures could improve their perceived engagement in patient care.

While the primary focus of this study was the PICU experience for residents, there are likely other factors which may have contributed to increased depression and burnout and which are less modifiable. The PICU may be residents’ first experience with the death of a patient, something variably experienced during first-year rotations. While residents did not identify patient acuity as a negative aspect of the rotation, mortality is somewhat separate from general acuity and could have a significant emotional impact. Additionally, for some residents, this rotation was their first time taking traditional 24-h call, which could have been an additional stressor not measured during this study. The impact of 24-h call on resident wellness has been studied in other environments but may confound these results.

There are several limitations to this study. In addition to the alternative explanation of these data already described, this study’s applicability is limited by the small sample size and single institution design. Due to the small sample size, statistical associations between aspects of the experience and depression and burnout, as well as individual changes in perception, could not be made. However, despite the small, single center study, many observations are likely true in other similarly-sized PICUs.

## Conclusions

The PICU experience is an important, potentially stressful, high acuity setting that can have a detrimental impact on the wellness of pediatric residents. However, given the high volume of pathology, there are many opportunities for education and development which can be captured. This study highlights room for improvements to the learning environment of the PICU. More thoughtful engagement of all members of the PICU team, including bedside nursing, residents, fellows and non-trainee team members, could lead to a more collaborative and productive environment. Follow-up studies on this population, as well as investigations of depression and burnout among other team members, could be useful in understanding and improving the PICU experience overall.

## Abbreviations

CES-D: Center for Epidemiologic Studies- Depression
PICU: Pediatric Intensive Care Unit
